# Assessment of Food and Drug Administration– and European Medicines Agency–Approved Systemic Oncology Therapies and Clinically Meaningful Improvements in Quality of Life: A Systematic Review

**DOI:** 10.1001/jamanetworkopen.2020.33004

**Published:** 2021-02-11

**Authors:** Vanessa Arciero, Seanthel Delos Santos, Liza Koshy, Amanda Rahmadian, Ronak Saluja, Louis Everest, Ambica Parmar, Kelvin K. W. Chan

**Affiliations:** 1 Evaluative Clinical Sciences, Odette Cancer Centre Research Program, Sunnybrook Research Institute, Toronto, Ontario, Canada; 2 Sunnybrook Odette Cancer Centre, University of Toronto, Toronto, Ontario, Canada; 3 Institute of Health Policy, Management and Evaluation, Dalla Lana School of Public Health, University of Toronto, Toronto, Ontario, Canada; 4 Canadian Centre for Applied Research in Cancer Control, Toronto, Ontario, Canada

## Abstract

**Importance:**

For patients with cancer treated with palliative intent, quality of life (QOL) is a critical aspect of treatment decision-making, alongside survival. However, regulatory approval can be based solely on survival measures or antitumor activities, without QOL evidence.

**Objective:**

To investigate whether recently approved oncology therapies demonstrate clinically meaningful improvements in QOL.

**Evidence Review:**

This systematic review study identified oncology drug indications approved by the US Food and Drug Administration (FDA) and European Medicines Agency (EMA) from January 2006 to December 2017 and supporting clinical trials (QOL publications identified to October 2019). Indications were evaluated for the presence of published QOL evidence; QOL benefits according to the American Society of Clinical Oncology Value Framework version 2.0 (ASCO-VF) and European Society of Medical Oncology Magnitude of Clinical Benefit Scale version 1.1 (ESMO-MCBS) QOL bonus criteria; and clinically meaningful improvements in QOL beyond minimal clinically important differences. Hematology trials were not evaluated by ESMO-MCBS. Associations between QOL evidence and approval year were examined using logistic regression models.

**Findings:**

In total, 214 FDA-approved (77 [36%] hematological) and 170 EMA-approved (52 [31%] hematological) indications were included. QOL evidence was published for 40% and 58% of FDA- and EMA-approved indications, respectively. QOL bonus criterion for ASCO-VF and ESMO-MCBS was met in 13% and 17% of FDA-approved and 21% and 24% of EMA-approved indications, respectively. Clinically meaningful improvements in QOL beyond minimal clinically important differences were noted in 6% and 11% of FDA- and EMA-approved indications, respectively. Availability of published QOL evidence at the time of approval increased over time for EMA (odds ratio [OR], 1.13; *P* = .03), however not for FDA (OR, 1.10; *P* = .12). Over time, no increase in awarded QOL bonuses or clinically meaningful improvements in QOL were found.

**Conclusions and Relevance:**

The findings of this systematic review suggest that approved systemic oncology therapies often do not have published evidence to suggest QOL improvement, despite its recognized importance. Of indications with evidence of statistical improvement, few have demonstrated clinically meaningful improvements.

## Key Points

**Question:**

Are oncology therapies recently approved by the US Food and Drug Administration (FDA) and European Medicines Agency (EMA) associated with clinically meaningful improvements in quality of life (QOL)?

**Findings:**

In this systematic review of approved oncology therapies, 40% of FDA-approved and 58% of EMA-approved indications had published QOL evidence. However, only 6% and 11% of FDA- and EMA-approved indications, respectively, had clinically meaningful improvements in QOL beyond minimal clinically important differences.

**Meaning:**

These findings suggest that oncology therapies are often approved without evidence of QOL improvement, despite the importance to patients.

## Teaser

This systematic review of US Food and Drug Administration and European Medicines Agency approvals for oncology therapies examines the prevalence of trials showing clinically meaningful improvements in quality of life as evidence in the approval process.

## Introduction

For patients with cancer treated with palliative intent, quality of life (QOL) is a critical aspect of treatment decision-making, alongside survival. In some cases, patients with advanced cancer have even prioritized QOL over survival.^1^ However, submission of QOL data are not mandatory for oncology drugs seeking regulatory approval by the US Food and Drug Administration (FDA) or European Medicines Agency (EMA). Thus, despite the value of QOL as a constituent of clinical benefit, it often does not appear to be a considerable factor in drug approval.^2^

Clinical trials with the aim of drug registration are designed with primary objectives focused on demonstrating traditional components of clinical benefit, such as overall survival (OS). As such, trials are therefore powered to capture the statistically significant differences in these traditional end points, often without consideration of QOL evidence. However, regulatory approval is frequently based on surrogate outcomes for survival or clinical effectiveness, such as progression-free survival (PFS) or response rate.^3,4,5^ While such methods may serve to expedite drug approval, a drug’s final efficacy (or the lack thereof) may not be apparent at the time of market authorization.^3,4,6^ Given an absence of clinically meaningful survival gains, any increase in length of life may not be associated with comparable increases in QOL.

The landscape of oncology therapeutics is rapidly evolving, but although moderate improvements in survival have been achieved, there is a paucity of literature surrounding clinically meaningful improvements in QOL for oncology drugs. While there has been growing attention to QOL end points^7,8^ and greater consideration of QOL within the concept of value in cancer care, there is seemingly little consideration of QOL by regulatory agencies.

In an attempt to quantify value and systematically assess the clinical benefit of oncology drugs, the American Society of Clinical Oncology (ASCO) and European Society of Medical Oncology (ESMO) have released valuation frameworks.^9,10^ Both frameworks intend to objectively quantify clinical benefit by considering outcome measures including survival, QOL, and toxic effects, and can be used to objectively evaluate the overall clinical benefit of oncology drugs or used in part to evaluate individual dimensions.

The ASCO Value Framework (ASCO-VF) and ESMO Magnitude of Clinical Benefit Scale (ESMO-MCBS) use a multidimensional approach to assess clinical benefit and, although initially developed for different purposes, may be used complementarily to evaluate the degree of clinical benefit using predefined thresholds.^11^ In order to assess QOL without consideration of survival or other constituents of clinical benefit overall, the QOL components of the ASCO-VF and ESMO-MCBS can be considered. ASCO-VF and ESMO-MCBS each assess QOL based on randomized clinical trial (RCT)–derived health-related QOL measures, such as those measured through the European Organization for Research and Treatment of Cancer (EORTC) and/or the reported profiles of toxic effects.^9,10^

While it is valuable to consider statistical significance for survival end points, patient-reported outcomes may inherently vary between time points irrespective of actual changes in the outcome.^12,13^ Thus, large trials may show statistically significant numerical differences in QOL that may not equate to clinically meaningful differences for patients.^12^ Therefore, it is also important to consider minimal clinically important difference (MCID) values when evaluating changes in QOL.

MCID values are defined as the smallest numerical difference in QOL measures that translates to clinically meaningful improvements for patients.^14^ MCID is recognized as an accepted method of interpreting and contextualizing QOL changes.^15^ Although meaningful changes to QOL can be subjective, MCID is a valuable concept in discerning the magnitude and clinical relevance, or lack thereof, of QOL improvements as evaluated by patients.^16^ MCID should be considered alongside measures for statistical significance, as comparable numerical changes in patient-reported outcomes may have various meanings across patient groups.^16^

Consideration of both QOL benefits as defined by valuation frameworks and MCID values is critical in assessing oncology therapeutics for QOL improvements. This study therefore aims to utilize such methods to investigate whether recently approved oncology therapies demonstrate clinically meaningful improvements in QOL.

## Methods

### Selection of Trials

We conducted a systematic review of trials cited as evidence for regulatory approval of systemic oncology therapies by the FDA (Hematology/Oncology Approvals and Safety Notification page^17^) and EMA (Public Assessment Reports^18^) between January 2006 and December 2017. Primary publications, supplementary appendices, and updated publications (to October 2019) were collected from Web of Science and ClinicalTrials.gov. Only full publications were reviewed. Trials reporting QOL evidence were considered for evaluation if in the noncurative setting. Data analysis was conducted until May 2020. Institutional review board approval was not necessary for the purposes of this project given all data were collected from published literature available in the public domain. This study followed the Preferred Reporting Items for Systematic Reviews and Meta-analyses (PRISMA) reporting guideline.

### Data Extraction

Trials were reviewed for eligibility by 2 reviewers (V.A. and S.D.), and key trial characteristics and data were extracted on standardized extraction templates, including trial characteristics (ie, National Clinical Trial number, publication year, phase, and treatment indication), systemic therapy, survival measures, and QOL data. Reported tools used to measure QOL were collected, alongside corresponding outcome data from primary and updated publications.

### Data Analysis/Synthesis

If QOL data were reported, we evaluated trials for QOL improvement using the ASCO-VF version 2.0^9^ and the ESMO-MCBS version 1.1^10^ QOL bonus criteria. In the ASCO-VF, a QOL bonus is assigned in instances where “a statistically significant improvement in treatment-free interval is reported for the regimen being evaluated,”^9^ whereas in the ESMO-MCBS a QOL bonus is assigned in instances where a “secondary end point QOL show[s] improvement” and/or “there [are] statistically significantly less grade 3-4 toxicities impacting daily well-being.”^10^ Given the varying approach to QOL among ASCO-VF and ESMO-MCBS, the accounting for toxic effects element of the ESMO-MCBS QOL bonus was not considered for the main data analysis in order to apply a more consistent approach to evaluating QOL. A sensitivity analysis considering the entirety of the ESMO-MCBS QOL bonus criterion was completed.

Scoring was completed by 2 authors using all publicly available data, with discrepancies resolved by consensus first or through consultation with a third author. When available, ASCO-VF QOL bonuses were collected from scores endorsed by ASCO,^11^ and ESMO-MCBS QOL bonuses collected from previously published scores on ESMO’s website.^19^ In concordance with the EMSO-MCBS framework, hematology trials were not scored using the EMSO-MCBS.

QOL data were also assessed using MCID values for each trial’s corresponding QOL assessment tool. If trials had self-reported MCID values, they were used to determine clinical meaningfulness. However, if an MCID value was not referenced by the trial, established MCID values for particular QOL assessment tools were identified (eTable in the Supplement). MCID values were used to evaluate clinically meaningful differences in QOL between the experimental and control arms of trials. Trials were deemed to demonstrate a clinically meaningful improvement in QOL if the QOL difference between arms met the MCID value. QOL changes were classified into categories: (1) improvement in QOL, (2) no improvement in QOL, or (3) deterioration in QOL.

Associations between QOL evidence (such as the presence or absence of published QOL evidence, ASCO-VF and/or ESMO-MCBS QOL bonuses being awarded, and clinically meaningful QOL based on MCID as binary dependent variables) and the FDA and EMA approval year (as independent variable) over time were then examined using logistic regression models in RStudio version 3.5.0 (R Project for Statistical Computing) with the trial phase modeled as an explanatory variable. The odds ratio (OR) for the availability of QOL evidence at FDA and EMA approval over time was computed separately.

The entirety of the ESMO-MCBS QOL bonus was evaluated in a sensitivity analysis, considering both (1) an improvement in QOL end point and/or (2) significantly fewer grade 3 to 4 toxic effects.^10^ The toxic effects component of the ESMO-MCBS QOL bonus considers grade 3 to 4 toxic effects impacting daily well-being (excluding alopecia and myelosuppression), such as chronic nausea, diarrhea, and fatigue, among others.^8^ Scoring for such sensitivity analysis was completed using the aforementioned method.

### Subgroup Analysis

Subgroup analyses were completed to investigate the presence of QOL evidence, QOL benefits according to ASCO-VF and ESMO-MCBS bonus criterion, and clinically meaningful improvements in QOL based on therapy type separately. Main subgroups included chemotherapy, targeted therapy, and immunotherapy.

### Risk of Bias

Risk of bias across trials informing regulatory approval with available QOL evidence was evaluated by 2 independent reviewers. Domains were categorized as low, high, or unclear risk based on all available evidence to our selected cutoff date, with a focus on QOL end points. Given that QOL evaluation is a patient-reported outcome, the most important sources of bias may be due to a lack of masking of participants and outcome assessors to treatment. Risk of bias data were aggregated using Review Manager 5.3 software (Cochrane Training) (eFigure 1 and eFigure 2 in the Supplement). A *P* value <.05 was considered significant in 2-sided tests.

## Results

### Characteristics of Included Trials

Two-hundred and fourteen FDA- and 170 EMA-approved indications were included. Of these, 77 FDA-approved indications (36%) and 52 EMA-approved indications (31%) were hematological malignant neoplasms. One-hundred and fourteen identified trials were cited as evidence for both FDA and EMA approvals. Characteristics of included indications are outlined in Table 1.

**Table 1.** Characteristics of Included Indications

Characteristic	Indications, No. (%)^a^	
	FDA approved (n = 214)	EMA approved (n = 170)
Phase		
I	5 (2.3)	2 (1.2)
I/II	1 (<1.0)	1 (<1.0)
II	60 (28.0)	26 (15.3)
II/III	0	1 (<1.0)
III	147 (68.7)	140 (82.4)
IV	1 (<1.0)	0
Disease site		
Genitourinary	21 (9.8)	18 (10.6)
Gastrointestinal	24 (11.2)	24 (14.1)
Breast	18 (8.4)	17 (10.0)
Hematologic	77 (36.0)	52 (30.6)
Lung	30 (14.0)	26 (15.3)
Gynecologic	7 (3.3)	7 (4.1)
Melanoma	14 (6.5)	11 (6.5)
Other	23 (10.7)	15 (8.8)
Therapy type		
Chemotherapy	30 (14.0)	26 (15.3)
Targeted therapy	142 (66.4)	118 (69.4)
Immunotherapy	32 (15.0)	18 (10.6)
Other	10 (4.7)	8 (4.7)

Abbreviations: EMA, European Medicines Agency; FDA, US Food and Drug Administration.

^a^ Percentages may not sum to 100% because of rounding.

**Figure 1.**
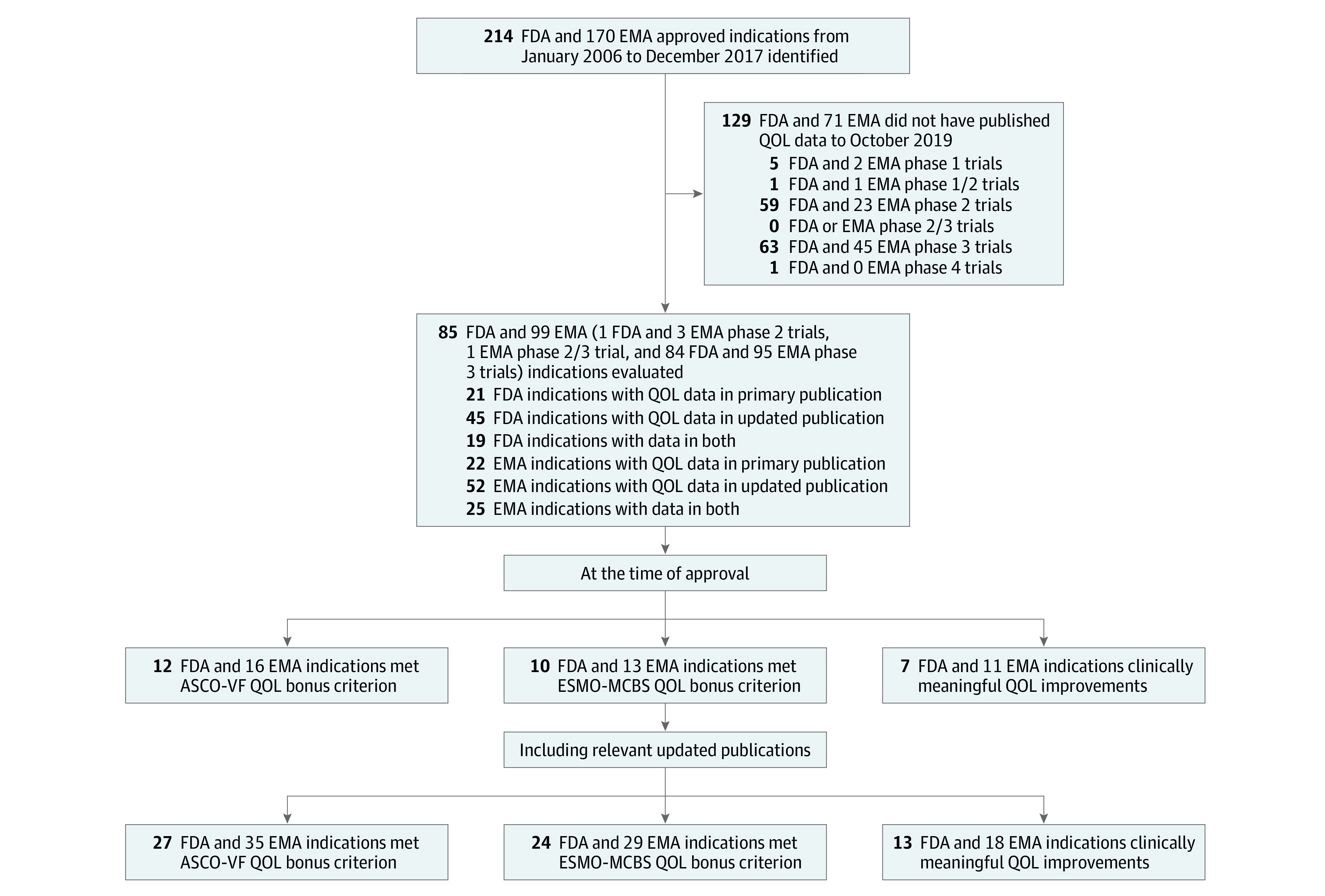
Flow Diagram of Included Indications ASCO-VF indicates American Society of Clinical Oncology Value Framework version 2.0; EMA, European Medicines Agency; ESMO-MCBS, European Society of Medical Oncology Magnitude of Clinical Benefit Scale version 1.1; FDA, US Food and Drug Administration; QOL, quality of life.

Published QOL evidence at the time of regulatory approval was available in 31 of 214 FDA-approved indications (14%) and 44 of 170 EMA-approved indications (26%). Considering all published QOL evidence to our selected cutoff date, 85 of 214 FDA-approved indications (40%) and 99 of 170 and EMA-approved indications (58%) had published QOL evidence (Figure 1). All indications with reported QOL evidence were in a noncurative setting.

Nearly all indications reporting QOL evidence (FDA, 83 of 85 [98%]; EMA, 97 of 99 [98%]) measured QOL using the Functional Assessment of Cancer Therapy (FACT) general or site-specific subscales, EuroQol (EQ-5D) family of questionnaires, and/or the EORTC subscales.

### ASCO-VF and ESMO-MCBS QOL Bonus

At the time of regulatory approval, 12 of 214 FDA-approved indications (6%) and 16 of 170 EMA-approved indications (9%) met the ASCO-VF QOL bonus criterion. Considering all published QOL evidence to our selected cutoff date, 27 of 214 FDA-approved indications (13%) and 35 of 170 EMA-approved indications (21%) met the ASCO-VF QOL bonus criterion.

**Figure 2.**
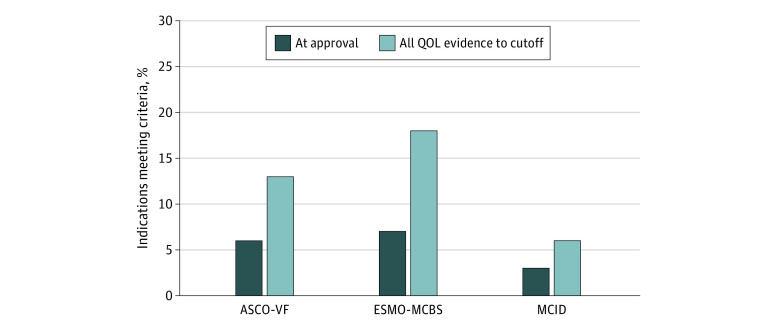
QOL Benefits and Clinically Meaningful Improvements for FDA-Approved Indications With Published QOL Data A total of 214 indications (77 hematological) were considered. European Society of Medical Oncology Magnitude of Clinical Benefit Scale version 1.1 (ESMO-MCBS) does not evaluate hematological malignant neoplasms (percentages based on solid tumors only). Selected cutoff date was October 2019. ASCO-VF indicates American Society of Clinical Oncology Value Framework version 2.0; FDA, US Food and Drug Administration; MCID, minimal clinically important difference; QOL, quality of life.

**Figure 3.**
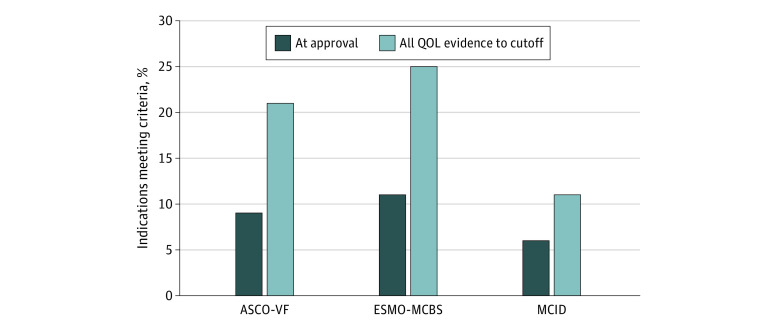
QOL Benefits and Clinically Meaningful Improvements for EMA-Approved Indications With Published QOL Data A total of 170 indications (52 hematological) were considered. European Society of Medical Oncology Magnitude of Clinical Benefit Scale version 1.1 (ESMO-MCBS) does not evaluate hematological malignant neoplasms (percentages based on solid tumors only). Selected cutoff date was October 2019. ASCO-VF indicates American Society of Clinical Oncology Value Framework version 2.0; EMA, European Medicines Agency; ESMO-MCBS, European Society of Medical Oncology Magnitude of Clinical Benefit Scale version 1.1; MCID, minimal clinically important difference; QOL, quality of life.

Excluding hematological malignant neoplasms (FDA, 77 [36%] indications; EMA, 52 [31%] indications), 10 of 137 FDA-approved indications (7%) and 13 of 118 EMA-approved indications (11%) met the ESMO-MCBS QOL bonus criterion at the time of regulatory approval. In comparison, when considering all published QOL evidence to October 2019, 24 of 137 FDA-approved indications (18%) and 29 of 118 EMA-approved indications (25%) met the ESMO-MCBS QOL bonus criterion (Figures 2 and 3).

FDA- and EMA-approved indications with published QOL evidence (excluding hematological malignant neoplasms) were also evaluated considering both elements of the ESMO-MCBS QOL bonus criterion. When considering all published QOL evidence to October 2019, only 3 additional FDA-approved and 3 EMA-approved indications met the MCBS bonus criterion.

Of 114 trials cited as evidence to inform both FDA and EMA approvals, 71 (62%) had QOL evidence published at the time of our selected cutoff date. ASCO-VF QOL criterion was met in 22 of 114 trials (19%) and ESMO-MCBS QOL criterion was met in 21 of 82 trials (26%) (excluding 32 hematological trials).

### MCID in QOL

At the time of regulatory approval, few FDA- and EMA-approved indications demonstrated clinically meaningful improvements in QOL beyond MCID (7 of 214 [3%] and 11 of 170 [6%], respectively). When considering all QOL evidence to October 2019 of approved FDA indications, clinically meaningful improvements in QOL beyond MCID were noted in only 13 of 214 indications (6%). Otherwise, 70 of 214 indications (33%) showed no clinically meaningful improvement in QOL, and 1 of 214 indications (0.5%) showed a deterioration in QOL. Similarly, of those indications approved by EMA, clinically meaningful improvements in QOL beyond MCID were noted in only 18 of 170 (11%). Nonetheless, 79 of 170 indications (46%) showed no clinically meaningful improvement in QOL, and 1 of 170 indications (0.6%) showed a deterioration in QOL (Figures 2 and 3).

### Time Trends

Multivariable models found no evidence of increasing QOL evidence published at the time of FDA approval over time (OR, 1.10; *P* = .12). Similarly, there were no significant associations between the increase in awarded QOL bonuses (ASCO-VF: OR, 0.98; *P* = .87; ESMO-MCBS: OR, 0.96; *P* = .77) or clinically meaningful improvements in QOL (OR, 0.92; *P* = .57) relative to the time of FDA approval were found over time.

A small signal was noted for increasing QOL evidence published at the time of EMA approval over time (OR, 1.13; *P* = .03). However, similar findings were not found for QOL bonuses (ASCO-VF: OR, 1.00; *P* = .96; ESMO-MCBS: OR, 1.03; *P* = .74) or clinically meaningful improvements in QOL (OR, 0.94; *P* = .56) for EMA approved indications over time.

### Subgroup Analysis

While a comparable percentage of indications had published QOL evidence across therapy type, numerically, targeted therapy and immunotherapy indications more frequently met QOL bonus criteria and clinically meaningful improvements in QOL relative to chemotherapy indications (Table 2).

**Table 2.** QOL Benefits and Clinically Meaningful Improvements Detailed by Therapy Type^a^

Therapy type	Total indications, No. (No. of hematological)	Indications, No. (%)			
		With QOL evidence	ASCO-VF QOL bonus	ESMO-MCBS QOL bonus^b^	MCID
**Chemotherapy**					
FDA	30 (13)	12 (40.0)	1 (3.3)	0 (0.0)	0 (0.0)
EMA	26 (8)	10 (38.5)	1 (3.8)	1 (5.6)	0 (0.0)
**Targeted therapy**					
FDA	142 (56)	61 (43.0)	16 (11.3)	14 (16.3)	8 (5.6)
EMA	118 (40)	73 (61.9)	24 (20.3)	17 (21.8)	14 (11.9)
**Immunotherapy**					
FDA	32 (5)	8 (25.0)	6 (18.8)	6 (22.2)	1 (3.1)
EMA	18 (3)	10 (55.6)	5 (27.8)	6 (40.0)	1 (5.6)
**Other**					
FDA	10 (3)	4 (40.0)	4 (40.0)	4 (57.1)	4 (40.0)
EMA	8 (1)	6 (75.0)	5 (62.5)	5 (71.4)	3 (37.5)

Abbreviations: ASCO-VF, American Society of Clinical Oncology Value Framework version 2.0; EMA, European Medicines Agency; ESMO-MCBS, European Society of Medical Oncology Magnitude of Clinical Benefit Scale version 1.1; FDA, US Food and Drug Administration; MCID, minimal clinically important difference; QOL, quality of life.

^a^ Considering all published QOL evidence to October 2019.

^b^ ESMO-MCBS does not evaluate hematological malignant neoplasms (percentages based on solid tumors only).

### Risk of Bias

Of approvals with QOL evidence, 33 of 85 (39%) FDA indications and 39 of 99 (39%) EMA indications cited double-masked trials, representing a low risk of performance and detection biases. The remaining trials were open-label (52 of 85 [61%] FDA and 60 of 99 [61%] EMA). Given that patients are not masked to the treatment assignment, this represents the potential for high risk of performance bias for the evaluation of the QOL end point toward an overestimation of the QOL benefit. Aggregated risk of bias data across all evaluated domains is presented in the eAppendix in the Supplement.

## Discussion

Despite the in-depth review of data by the FDA and EMA for drugs seeking regulatory approval, the majority of approvals are based on survival end points, with limited published QOL evidence available at the time of approval.^20,21^ While 40% of FDA and 58% of EMA approved indications evaluated in our study had publicly available QOL evidence, few indications met the ASCO-VF and/or ESMO-MCBS QOL bonus criteria (13% FDA and 21% EMA approvals, and 18% FDA and 25% EMA approvals, respectively), while even fewer met clinically meaningful improvements in QOL beyond MCID (6% FDA and 11% EMA approvals). Our results suggest that while few recently approved FDA and EMA oncology drugs have published evidence to suggest QOL improvement, even fewer of those that do show statistical improvement have evidence of clinically meaningful improvements.

There are numerous aspects of clinical benefit to consider when evaluating a drug seeking regulatory approval, including survival, QOL, toxicity, and strength of evidence. While end points that are of greatest value to patients should be of highest consideration by regulatory boards, our findings highlight a disconnect between what is meaningful and important to patients and what is being favorably approved by regulatory boards. Nonetheless, it should be noted that QOL measures may be inherently subjective in measurement, especially in open-label randomized trials, and vary widely across a population. Therefore a standardized approach to QOL evaluation across all trials designed to seek approval from regulatory agencies presents challenges.^22^

While regulatory approvals are most frequently based on promising findings in surrogate end points for survival, of the 71 solid-tumor drugs approved by the FDA between 2002 and 2014, there were only modest improvements in median PFS and OS (2.5 and 2.1 months, respectively).^23^ A plethora of previously published work suggests that, overall, recently approved oncology drugs demonstrate limited clinically meaningful benefits based on total ASCO-VF and ESMO-MCBS scores.^24,25,26,27^ Of note, Saluja et al^27^ suggest that such marginal improvements in overall clinical benefit as measured by the ASCO-VF and ESMO-MCBS are accompanied by substantial increases in cost over time. Our findings serve to extend previously published work suggesting that, when considering QOL alone, a fairly limited subset of recently approved oncology drugs achieve differences in QOL that can be considered clinically meaningful. These findings are especially concerning for patients with advanced disease. As the goals of care may evolve throughout the course of disease, QOL may become of greater importance for those treated with palliative intent.^1^

The ESMO-MCBS states that the QOL bonus may be assigned where a “secondary end point QOL show[s] improvement” and/or “there [are] statistically significantly less grade 3-4 toxicities impacting daily well-being.”^10^ Given the complex and multidimensional aspects involved in QOL and its appropriate collection and evaluation, we elected to focus on the first component of such bonus for the primary analysis in order to offer concordance with the ASCO-VF version 2.0.^9^ Nonetheless, sensitivity analyses investigating both components of the ESMO-MCBS QOL bonus did not yield largely differing results. The toxicity profile is an important component in QOL evaluation given data suggesting QOL measures often do not capture adverse events.^28,29,30^ These findings further emphasize the number of measures of patient-relevant end points that are not adequately captured at the time of regulatory approval.

In order to assess eligibility for QOL bonuses and to align with the intent of how QOL bonus would be scored by the developers of ASCO-VF and ESMO-MCBS, when available, ASCO-VF QOL bonuses were collected from scores endorsed by ASCO,^11^ and ESMO-MCBS QOL bonuses collected from ESMO’s website.^19^ The remaining scores were completed by 2 independent reviewers, with consultation of a third reviewer when necessary, with experience applying the valuation frameworks.^6,27,31,32,33,34,35^

### Limitations

This study had several limitations. Patients enrolled in clinical trials are often highly selected compared with the typical oncology population. Specifically, older patients, those with multiple comorbidities, or patients with more complex cases may not be eligible to participate in clinical trials. Additionally, our study is based on publicly available evidence from the FDA, EMA, and informing trials. Despite our rigorous review, regulatory agencies may have access to additional evidence from trials that could be used to inform approvals beyond what is publicly available. The inability to assess heterogeneity in patient expectations surrounding the appropriate balance between quality and quantity of life is an additional limitation. Literature suggests that older patients may preferentially value QOL over quantity while younger patients may prefer quantity of life.^36^ Although this could not be assessed, our results highlight the need for QOL evidence alongside true efficacy to better inform individualized discussions surrounding patient preferences.

QOL data are inherently more difficult to collect and evaluate than survival outcomes.^22^ While our findings highlight the paucity of available QOL evidence overall in recently approved oncology drugs, and that even fewer indications offer statistically and clinically meaningful improvements in QOL, we recognize the limitations within individual trials to collect and analyze such data in meaningful ways.

## Conclusions

Our study suggests that systemic oncology therapies are often approved by regulatory agencies without evidence to demonstrate QOL improvement. Notably, of those indications with evidence of statistical improvement in QOL, our results suggest that limited indications also have evidence of clinically meaningful improvements. Given the high value patients place on QOL end points, the scarcity of available and favorable QOL evidence for approved systemic oncology therapies should be recognized. This is especially important in the context of systemic therapy with palliative intent, as the goals of therapy may evolve over the course of disease to prioritize QOL over quantity of life.^1^

RCTs should be encouraged to not only improve collection and reporting of QOL evidence, but to do so in a manner that provides informative evidence of meaningful clinical benefit, to aid in regulatory approval and clinical decision-making that best supports patient-centered care. In addition, regulatory agencies should seek to enhance consideration of QOL evidence as a more considerable constituent of their approval processes and requirements, to reflect evolving patient priorities.

## Notes

Supplement. **eTable****.** Values Used to Denote a Clinically Meaningful Change in QOL Across Various QOL Assessment Tools **eFigure 1.** Risk of Bias Across Trials Informing Food and Drug Administration-Approval With Available Quality of Life Evidence (n=85) **eFigure 2.** Risk of Bias Across Trials Informing European Medicines Agency-Approval With Available Quality of Life Evidence (n=99) **eReferences**
