# Supplementary material for: Assessment of Food and Drug Administration– and European Medicines Agency–Approved Systemic Oncology Therapies and Clinically Meaningful Improvements in Quality of Life: A Systematic Review
Source: JAMA Netw Open. 2021 Feb 11;4(2):e2033004. doi: 10.1001/jamanetworkopen.2020.33004 (PMC7879236; doi:10.1001/jamanetworkopen.2020.33004)
Supplement: Supplement. — eTable. Values Used to Denote a Clinically Meaningful Change in QOL Across Various QOL Assessment Tools eFigure 1. Risk of Bias Across Trials Informing Food and Drug Administration-Approval With Available Quality of Life Evidence (n=85) eFigure 2. Risk of Bias Across Trials Informing European Medicines Agency-Approval With Available Quality of Life Evidence (n=99) eReferences [file jamanetwopen-e2033004-s001.pdf]

Supplemental Online Content

Arciero V, Delos Santos S, Koshy L, et al. Assessment of Food and Drug Administration– and European Medicines Agency–approved systemic oncology therapies and clinically meaningful improvements in quality of life. *JAMA Netw Open*. 2021;4(2):e2033004. doi:10.1001/jamanetworkopen.2020.33004

eTable. Values Used to Denote a Clinically Meaningful Change in QOL Across Various QOL Assessment Tools
eFigure 1. Risk of Bias Across Trials Informing Food and Drug Administration-Approval With Available Quality of Life Evidence (n=85)
eFigure 2. Risk of Bias Across Trials Informing European Medicines Agency-Approval With Available Quality of Life Evidence (n=99)
eReferences

This supplemental material has been provided by the authors to give readers additional information about their work.

© 2021 Arciero V et al. *JAMA Network Open.*

eTable1: Values Used to Denote a Clinically Meaningful Change in QOL Across Various QOL Assessment Tools

Tool	MCID Values
EQ-5D Index	0.08 points[1]
EQ-Visual Analogue Scale (-VAS)	7 points[1]
EORTC Quality of Life Questionnaire (QLQ-C30)	10 points in global health status/QOL[2]
EORTC QLQ Head and Neck Cancer (H&N 35)	10 points[3]
EORTC QLQ Breast Cancer (BR23)	10 points[4]
EORTC QLQ Myeloma (MY20)	10 points - disease symptoms<br>6 points - side effects[5,6]
FACT-General (-G) Total	3-7 points[7]
FACT-Prostate (-P) Total	6-10 points[8]
FACT-P Trial Outcome Index (TOI)	5-9 points[8]
FACT-Breast (-B) Total	7-8 points[9]
FACT-B TOI	5-6 points[9]
FACT-Lymphoma (-Lym) Total	7 points[10]
FACT-Lung (-L) TOI	5-6 points[11]
FACT-L Lung Cancer Subscale (LCS)	2-3 points[11]
FACT-Anemia (-An) Total	7 points[11]
FACT-Gastric (-Ga)	15.1 points – small change<br>22.6 points – large change[12]
FACT-Ovarian (-O)	9 points[13]
FACT-O TOI	7 points[13]
Functional Ovarian Symptom Index (FOSI)	3 points[13]
FACT-Hepatobiliary (-Hep)	8-9 points[14]
FACT-Cervix (-Cx) TOI	5.8-8.7 points[7]
FACT Kidney Cancer Symptom Index Disease Related Symptoms (FKSI-DRS)	2-3 points[15]
FKSI-15 Item Version (-15)	3-5 points[16]
FACT Breast Symptom Index (FBSI)	2-3 points[17]
Lung Cancer Symptom Scale (LCSS)	15 mm change from baseline[18]
Brief Pain Inventory (BPI)	2 points[19]
Patient-Reported Outcomes Measurement Information System - Cancer (PROMIS-Cancer) (7 item)	3-5 points[20]

EORTC: European Organization for Research and Treatment of Cancer; EQ-5D: EuroQol; FACT: Functional Assessment of Cancer Therapy; MCID: minimal clinically important difference; QOL: quality of life

© 2021 Arciero V et al. *JAMA Network Open.*

Figure 1. Risk of bias across trials informing Food and Drug Administration-approval with available quality of life evidence (n=85)*

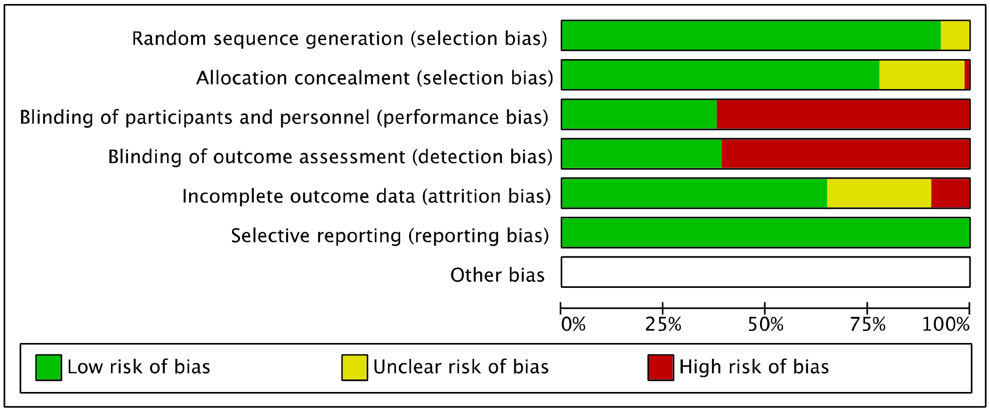

*Review Manager (RevMan) [Computer program]. Version 5.3. Copenhagen: The Nordic Cochrane Centre, The Cochrane Collaboration, 2014 was used to aggregated risk of bias data

© 2021 Arciero V et al. *JAMA Network Open.*

eFigure 2. Risk of bias across trials informing European Medicines Agency-approval with available quality of life evidence (n=99)*

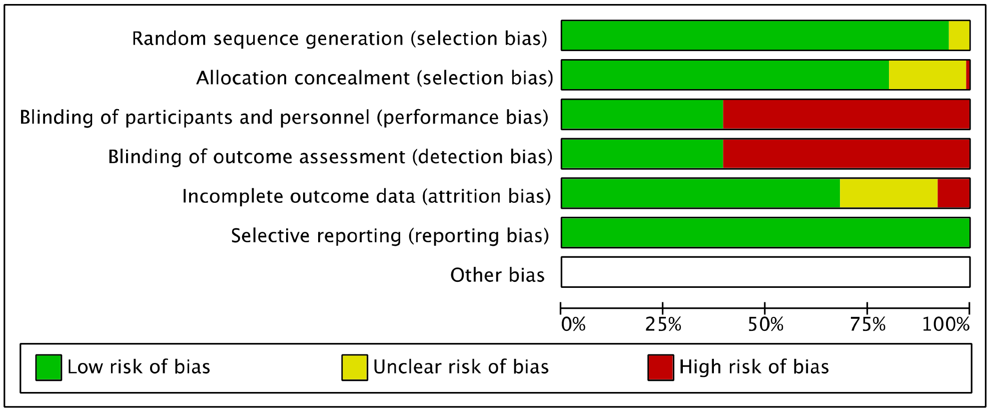

*Review Manager (RevMan) [Computer program]. Version 5.3. Copenhagen: The Nordic Cochrane Centre, The Cochrane Collaboration, 2014 was used to aggregated risk of bias data

© 2021 Arciero V et al. *JAMA Network Open.*

eReferences

1. Pickard AS, Neary MP, Cella D. Estimation of minimally important differences in EQ-5D utility and VAS scores in cancer. *HQOL.* 2007;5(1):70.

2. Osoba D, Rodrigues G, Myles J, et al. Interpreting the significance of changes in health-related quality-of-life scores. *JCO.* 1998;16(1):139-44.

3. Bottomley A, Tridello G, Coens C, et al. An international phase 3 trial in head and neck cancer: quality of life and symptom results: EORTC 24954 on behalf of the EORTC Head and Neck and the EORTC Radiation Oncology Group. *Cancer*. 2014;120(3):390-8.

4. Osoba D, Slamon DJ, Burchmore M, et al. Effects on quality of life of combined trastuzumab and chemotherapy in women with metastatic breast cancer. *JCO*. 2002;20(14):3106-13.

5. Delforge M, Minuk L, Eisenmann JC, et al. Health-related quality-of-life in patients with newly diagnosed multiple myeloma in the FIRST trial: lenalidomide plus low-dose dexamethasone versus melphalan, prednisone, thalidomide. *Haematologica*. 2015;100(6):826-33.

6. Dimopoulos MA, Delforge M, Hájek R, et al. Lenalidomide, melphalan, and prednisone, followed by lenalidomide maintenance, improves health-related quality of life in newly

© 2021 Arciero V et al. *JAMA Network Open.*

diagnosed multiple myeloma patients aged 65 years or older: results of a randomized phase III trial. *Haematologica*. 2013;98(5):784-8.

7. Yost KJ, Eton DT. Combining distribution-and anchor-based approaches to determine minimally important differences: the FACIT experience. *Eval Health Prof*. 2005;28(2):172-91.

8. Cella D, Nichol MB, Eton D, et al. Estimating clinically meaningful changes for the Functional Assessment of Cancer Therapy—Prostate: results from a clinical trial of patients with metastatic hormone-refractory prostate cancer. *Value Health*. 2009;12(1):124-9.

9. Eton DT, Cella D, Yost KJ, et al. A combination of distribution-and anchor-based approaches determined minimally important differences (MIDs) for four endpoints in a breast cancer scale. *J Clin Epidemiol*. 2004;57(9):898-910.

10. Cheson BD, Trask PC, Gribben JG, et al. Health-related quality of life and symptoms in patients with rituximab-refractory indolent non-Hodgkin lymphoma treated in the phase III GADOLIN study with obinutuzumab plus bendamustine versus bendamustine alone. *Ann Hematol*. 2017;96(2):253-9.

11. Cella D, Eton DT, Fairclough DL, et al. What is a clinically meaningful change on the functional assessment of Cancer therapy–lung (FACT-L) questionnaire?: results from eastern cooperative oncology group (ECOG) study 5592. *J Clin Epidemiol*. 2002;55(3):285-95.

© 2021 Arciero V et al. *JAMA Network Open.*

12. Garland SN, Pelletier G, Lawe A, et al. Prospective evaluation of the reliability, validity, and minimally important difference of the functional assessment of cancer therapy-gastric (FACT-Ga) quality-of-life instrument. *Cancer*. 2011;117(6):1302-12.

13. Ledermann JA, Harter P, Gourley C, et al. Quality of life during olaparib maintenance therapy in platinum-sensitive relapsed serous ovarian cancer. *BJC*. 2016;115(11):1313-20.

14. Steel JL, Eton DT, Cella D, et al. Clinically meaningful changes in health-related quality of life in patients diagnosed with hepatobiliary carcinoma. *Ann Oncol*. 2006;17(2):304-12.

15. Cella D, Yount S, Brucker PS, et al. Development and validation of a scale to measure disease-related symptoms of kidney cancer. *Value Health*. 2007;10(4):285-93.

16. Cella D, Yount S, Du H, et al. Development and validation of the functional assessment of cancer therapy-kidney symptom index (FKSI). *J Support Oncol.* 2006;4(4):191-9.

17. Yost KJ, Yount SE, Eton DT, Silberman C, Broughton-Heyes A, Cella D. Validation of the functional assessment of cancer therapy-breast symptom index (FBSI). *Breast Cancer Res Treat*. 2005 Apr;90(3):295-8.

© 2021 Arciero V et al. *JAMA Network Open.*

18. De Marinis F, Pereira JR, Fossella F, et al. Lung Cancer Symptom Scale outcomes in relation to standard efficacy measures: an analysis of the phase III study of pemetrexed versus docetaxel in advanced non-small cell lung cancer. *JTO*. 2008;3(1):30-6.

19. Mathias SD, Crosby RD, Qian Y, et al. Estimating minimally important differences for the worst pain rating of the Brief Pain Inventory-Short Form. *J Support Oncol*. 2011;9(2):72-8.

20. Yost KJ, Eton DT, Garcia SF, et al. Minimally important differences were estimated for six Patient-Reported Outcomes Measurement Information System-Cancer scales in advanced-stage cancer patients. *J Clin Epidemiol*. 2011;64(5):507-16.

© 2021 Arciero V et al. *JAMA Network Open.*
